# Late fertility intentions increase over time in Austria, but chances to have a child at later ages remain low

**DOI:** 10.1016/j.rbms.2021.10.002

**Published:** 2021-11-23

**Authors:** Éva Beaujouan

**Affiliations:** Wittgenstien Centre for Global Human Capital, University of Vienna, Vienna, Austria

**Keywords:** Late parenthood, Childbearing postponement, Fertility intentions, Reproductive aging, Low-fertility countries

## Abstract

Childbearing takes place at increasingly older ages, and fertility is continuing to decrease across female birth cohorts. This study investigated whether the proportion of women who unintentionally forwent childbearing increased over time, and linked this to the age profile of fertility intentions and realization among men and women. This study was based on the Austrian Micro-Censuses (1986–2016) and on the Austrian Generations and Gender Surveys (panel data 2008/09 and 2012/13). Across the birth cohorts 1950–1979, an increasing proportion of women wanted to have children after 40 years of age, but more women failed to meet their fertility intentions expressed at 34–36 years of age. At the individual level, from 30 years of age, more than one-third of women and men with a strong fertility intention were found to persist with this intention within four years even at less fertile ages. In addition, women and men with a strong fertility intention became less likely to have a child with age: <10% of women and approximately 20% of men who had expressed a certain and short-term intention to have a child at 39–41 years of age in 2008/09 had a child by 2012/13. In particular, childless women and men, and those with only one child, persisted in certain and short-term positive intentions from 30 years of age, but parity was not a significant factor in their realization. The sharp increase in ‘unrealized fertility’ over time draws attention to the importance that personal circumstances and context encountered at older ages may have for fertility, and augurs a continued increase in the use of assisted reproduction.

## Introduction

In all European countries, having children, whether the first child or subsequent children, relies increasingly on later reproductive life (Beaujouan, 2020, Billari et al., 2007, Prioux, 2005). Delayed parenthood means higher risk of sterility and recourse to assisted reproduction (Ben Messaoud et al., 2020, Leridon, 2004, O’Brien and Wingfield, 2018), implying that as more people try to have a child later, more people also fail. The strong rise in extremely late childbirth also suggests that assisted reproductive technology (ART) and alternative procedures (e.g. egg donation) are becoming more common in the making of a family at an advanced maternal age (Beaujouan, 2020). The experience of infertility and involuntary childlessness, as well as repeated assisted reproductive treatments, are often associated with psychological and emotional distress among women and, to a lesser extent, among men (Doyle and Carballedo, 2014, Greil et al., 2010). Therefore, whether more people see their fertility plans constrained today in countries where fertility is delayed is an important reproductive health issue. Studying the relationship between fertility intentions and fertility realization at later ages will provide insights into the proportion of women and men potentially facing obstacles to having children.

Childlessness has become more prevalent, and the number of children that people have over their lifetime has decreased continuously (Sobotka, 2017, Sobotka et al., 2011). Such a trend is usually attributed to the decades-long change in childbearing norms and values (Lesthaeghe, 2010), but a small part of it has also been attributed to increasing sterility with age (Leridon and Slama, 2008, te Velde et al., 2012). A debate is ongoing regarding the extent to which childlessness is voluntary or involuntary, and – by extension – whether women and men have all the children they wish (Keizer et al., 2008, Letherby, 2002, Mynarska et al., 2015). Within a country, the proportion of people who do not have a child results from a ‘specific combination of intended childlessness, postponed decisions leading to involuntary childlessness, or constraints affecting abilities to achieve intentions at the individual level’ (Fiori et al., 2017: 319; see also Buhr and Huinink, 2017). To better understand the experience of infertility and sterility, as well as increasing ART needs due to fertility postponement, it is essential to better identify the involuntary part of childlessness, and unrealized fertility at higher parities.

Austria is in the mid-range of Western European countries in terms of later fertility: mothers aged ≥ 35 years contribute 22% of the total fertility rate, when the contribution is, in general, more than one-fifth across these countries (author’s calculations based on the Human Fertility Database). While first births at ≥ 35 years of age were limited to 5% of the total first birth rate in the middle of the 20th century, they reached 15% in 2016. Childlessness also increased quickly, and with 20% of women born in 1978 being childless, rates are closest to the Western European countries, above Eastern European countries but below Southern European countries (VID and IIASA, 2020).

This study first investigated changes in late aggregate fertility intentions and childbearing in Austria over the last 30 years using the Austrian Micro-Censuses (1986–2016). In addition, cohort trends in late positive intentions to have children (around 35 years of age) and the associated ‘fertility gap’ at the population level were explored among women. Such studies give a global estimation of ‘unrealized fertility’. This was complemented by examination of the importance of age in realizing or conserving strong short-term fertility intentions among men and women, using the retrospective and panel features of the Austrian Generations and Gender Surveys (GGS 2008/09 and 2012/13). Finally, realization and persistence of strong short-term intentions from 30 years of age onwards were modelled, controlling for parity, partnership status and level of education. This way, the factors that can constrain or favour the fertility of men and women at older ages were identified, and revealed the characteristics of those who persist with their wish for children as they reach less fertile ages, and hence who are most likely to need access to assisted reproduction.

## Background

In the last quarter of the 20th century, most women in high-income countries were having their children in their 20 s, so the question of how late they could wait affected very few of them. Today, however, new questions have arisen due to increasingly late birth schedules. Some people (called ‘perpetual postponers’) continue wanting to have a child at ages when having a child is unlikely, regardless of whether or not they have tried to have a child previously (Berrington, 2004). Permanent inability to have a child increases rapidly from 35 years of age, and at 40 years of age, 35–45% of women who start trying to conceive will not have a child at all (Leridon, 2004, Menken and Larsen, 1986). Having waited too long can thus result in not having the child(ren) one intended, because one never had the opportunity to try or directly because of the experience of infertility (Koert and Daniluk, 2017). The present study investigated ‘unrealized fertility’, which covers those of any parity (with or without children) who wish to have a child but will not have one (Casterline and Han, 2017).

Studies of fertility intentions are not conclusive about the prevalence of unrealized fertility at the country level. In Italy in 2009, the proportion of childless women who wanted a child at 35–39 years of age was 64% and the proportion of childless women who had a child from that age was 31% (Beaujouan and Sobotka, 2017). In Austria in 2012, the proportions were 50% and 21%, respectively. The contrast was much greater at 40–44 years of age, but was much smaller among women who already had children, particularly two or more. In the USA at 40 years of age, 15% of women born around 1960 were childless, and 22% of them still wanted to have a child (Rybińska and Morgan, 2019). Few of these women had children (3% of the childless) by 46 years of age and 8% still wanted to have a child. Finally, in Australia, only 67% of men and 48% of women aged 35–37 years with strong fertility intentions in 2011 had a child within 4 years, and the respective figures for those aged ≥ 38 years were 52% and 24% (Beaujouan et al., 2019). These figures show a fairly strong contrast between the desire for children and childbearing itself at older ages, which suggests sizeable unrealized fertility.

Even less is known about changes over time. While childbearing is being postponed, one can expect that the proportion of people who experience obstacles to their fertility intentions as well as unrealized fertility will rise. Using micro-simulation, Te Velde et al. (2012) estimated that involuntary childlessness doubled in six European countries between the 1970s and 2000s. Another sign that the proportion of women experiencing fertility issues at older ages may be rising is that the use of infertility treatment at ≥ 35 years of age has risen quickly over the last decade, and more than at younger ages [Ben Messaoud et al. (2020) for France]. Across Europe, 50.5% of women treated with in-vitro fertilization were aged ≥ 35 years in 2005, compared with 54.2% in 2015 (the countries included in 2015 differ somewhat, but keeping the same countries as in 2005 would increase the figure even further) (Andersen et al., 2009, De Geyter et al., 2020). Such an increase does not necessarily mean that more women are experiencing biological constraints at older ages, but may simply indicate that more women tried ART after they had reached 35 years of age: help-seeking behaviour may have changed across ages. To better capture the effects of childbearing postponement, *the first aim of this study was to gauge the level of unrealized fertility in the population and whether it has changed over time, based on an empirical case study in Austria*.

Unrealized fertility can also be measured by following people’s intentions to have children and childbirths in the later stages of their reproductive life. Fertility intentions are volatile and change across the life course, and this can be particularly true when women and men near the end of their reproductive years (Beaujouan et al., 2019, Buhr and Huinink, 2017, Gemmill, 2018, Gray et al., 2013, Rybińska and Morgan, 2019). Observing such individual variations complements the aggregate picture of unrealized fertility. In addition, collecting information about men is particularly important because research on infertility still focuses primarily on women. Men aged > 35 years who wish to have children have them more frequently than women (Beaujouan et al., 2019). On the one hand, men are less subject to social, cultural and biological limitations in terms of timing of childbearing compared with women (Beaujouan and Solaz, 2013, Billari et al., 2011, Eisenberg and Meldrum, 2017, Fisch and Braun, 2005, de La Rochebrochard et al., 2006), and the age difference with the partner tends to increase with age at partnership formation (Beaujouan, 2011, Bozon, 1990). On the other hand, like women, men delay parenthood and become more exposed to their own or their partner’s inability to have children as they age. For instance, although men still have children above age 45 more often than women, the male–female difference is decreasing as fertility is being postponed (Beaujouan, 2020). *The second aim of this study was therefore to refine the aggregate view of unrealized fertility by studying the mechanisms of change in intentions, and their realization in later reproductive life among women and men*. After a brief description of the fertility intentions of women and men in Austria across ages, particularly in their 30 s and early 40 s, the proportion who actually had a child, maintained the same fertility intention or changed their fertility intention within 4 years was investigated

Realization of fertility intentions as well as their persistence depend on many factors. Mothers plan a birth less often than childless women, but parents realize short-term positive childbearing intentions more often than those without children (Dommermuth et al., 2015, Harknett and Hartnett, 2014). Also, the lower the parity, the more likely it is that an individual will persist with their wish to have a child (Kapitány and Spéder, 2012). Partnership status is central to fertility intentions, and those who live with a partner, whether married or cohabiting, usually display higher and more certain intentions (Beaujouan et al., 2019, Gray et al., 2013, Hayford, 2009, Iacovou and Patricio Tavares, 2011, Liefbroer, 2009). They are more likely to have their intended child(ren) than unpartnered people, and are more likely to persist with a positive intention (Kapitány and Spéder, 2012, Spéder and Kapitány, 2013). Finally, highly educated women delay childbearing more and are more often childless (Lappegård and Rønsen, 2005, Neels et al., 2017). In the studies available, after accounting for parity and age, short-term realization depends little on educational level (Pailhé and Régnier-Loilier, 2017, Spéder and Kapitány, 2013). However, highly educated women appear to be less likely than their lower educated counterparts to renounce having a child within 3 years (Kapitány and Spéder, 2012). From these studies, it seems likely that the realization of fertility intentions and their persistence will also depend on people’s characteristics at later ages.

*The third and final aim of this study was to understand the characteristics of older men and women who realize or do not realize their certain short-term positive fertility intentions*. We particularly explored which people persisted with a positive fertility intention without having children despite decreasing chances of being able to have a child. Unlike previous studies that covered all age ranges (Beaujouan et al., 2019), the present study focused solely on women and men aged ≥ 30 years in order to better understand the mechanisms in later reproductive life and the characteristics of those who could potentially need assisted reproduction. Indeed, the use of infertility treatment is now highest in people in their 30s (Ben Messaoud et al., 2020), and more information about the factors of fertility realization at that age are necessary to better target people who may need assistance. This study will also provide complementary information for individual studies of involuntary childlessness (Buhr and Huinink, 2017, Gemmill, 2018, Rybińska and Morgan, 2019).

## Materials and methods

### Data

#### Fertility intentions

In Austria, fertility intention questions are captured in two important data sources: the Austrian Micro-Census (every 5 years from 1986 until 2016), which is used for a description of trends at the country level; and the Generation and Gender Surveys (GGS: wave 1 in 2008/09 and wave 2 in 2012/13), which is used to better understand changes in intentions in individuals across ages.

Austrian Micro-Census data enable time series of female fertility intentions to be drawn. Indeed, the same question was asked over time, and pre-codes of this question did not change. Women aged 20–45 years were asked about their fertility intentions, but this was restricted to women aged < 40 years in 2006. Given the nature of fertility intention questions, proxy answers were dropped (i.e. answers that were given by another member of the household when the person targeted was absent). Each year, this represented between 20% and 30% of all the people interviewed in the age group selected. Depending on the year, between 2686 and 6613 women were thus asked about their fertility intentions in the Micro-Census. The question used in the current study translates in English to: ‘Do you wish to have one or several (additional) child at some point in your life? Please count ongoing pregnancies’. Possible answers were: 'yes', 'no' and 'don’t know'. This question was used to calculate the proportion of women who do not wish to have a child at a given age. The total number of children intended was calculated by summing the number of additional children wished by the respondent (asked in the following question of the micro-census) with the number of children that she already had.

The first wave of the GGS took place in Austria in 2008/09, with face-to-face interviews with a sample of men and women representative of the Austrian population. A few filters applied (see also Beaujouan, 2014), so that 1781 men and 2711 women aged 18–45 years were eventually asked the questions on fertility intentions. The second wave (2012/13) included the same fertility intention questions, and available data allow reconstruction of the partnership and fertility events that took place between the waves. Due to attrition, in total, 1132 men and 1871 women were interviewed in the two waves and had data available to study realization of, and change in, fertility intentions.

The main questions on intentions are translated as: (1) ‘Do you want an (additional) child now?’ (possible answers: ‘yes’, ‘no’, don’t know); (2) ‘Do you intend to have a child in the next 3 years?’ (possible answers: ‘certainly not’, ‘probably not’, ‘probably yes’ and ‘certainly yes’); and (3) ‘If you don’t have a child in the next 3 years, do you want one afterwards?’ (possible answers: ‘certainly not’, ‘probably not’, ‘probably yes’ and ‘certainly yes’).

This study adopted a narrow definition of positive intentions (i.e. certainly wishing to have a child within 3 years), termed ‘strong intention to have a child’. This was to try to circumvent uncertainty and to narrow down the range to those who were most likely to try to have a child before the next wave. Accordingly, the intention variable was constructed the following way: people who want a child right now (Question 1) or within 3 years and are certain about their positive intention (Question 2) constitute the category ‘yes, very sure’. All the other people with positive intentions were classified as ‘yes, unsure’. All those with negative intentions were classified as ‘no more’. The second wave was then used to determine whether the respondents had a child between the waves, as well as their fertility intention at the time of the second wave if they did not have a child.

#### Other variables for the longitudinal study of intentions in GGS

'Age' is the age of the respondent at the time of the first wave. ‘Parity’ is based on the number of children that the individual declared in the first wave. ‘Level of education’ was constructed based on the highest diploma obtained in the first wave, and recoded using the ISCED 1997 scale: low 0–2 (no education, lower secondary), medium 3–4 (higher secondary) and high 5–6 (university degree, higher education). Finally, ‘partnership situation’ was controlled for in the first wave, differentiating between ‘married’, ‘partner unmarried’ (in an unmarried cohabiting partnership) and ‘no partner’ (no cohabiting partner).

### Methods

In the first instance, the change in aggregate intentions over time was analysed at 35–39, 40–42 and 43–44 years of age in the Micro-Censuses. In addition, the gap between fertility intentions around 35 years of age and actual fertility at the aggregate level in cohorts of women born between 1950 and 1974 was evaluated. To do this, the Micro-Censuses were pooled, mean intended family size at 34–36 years of age and mean completed family size (i.e. average number of children at 40–49 years of age) within the same cohort groups were calculated, and the difference was calculated. The same was done for the proportion of individuals who were eventually childless and the proportion who did not intend to have a child to estimate the share who were ‘unwillingly childless’. The indicators were weighted using survey weights to account for survey design and non-response.

To better understand aggregate intentions in later reproductive life, an overview is given of fertility intentions by age, and how they are realized or renounced depending on that age, for both men and women and using the panel feature of the Austrian GGS. Parity is key to the variation in childbearing intentions with age, so the intention age profiles are displayed by parity. Next, the factors of realization of strong positive fertility intentions and change in intentions are explored. The age profile of fertility intentions by parity in the first wave of GGS is shown, and then the second wave of GGS is used to display the age profiles of realization and change in intentions between 2008/09 and 2012/13 depending on original intentions (‘yes, very sure’, ‘yes, unsure’ and ‘no more’).

Finally, the factors of realization or change in fertility intentions among the respondents with strong initial intentions (as defined above) were studied. In this study of later intentions, 176 men and 186 women aged 30–41 years were selected, as justified theoretically above. Multinomial models were estimated separately for men and women with three outcomes [birth or (partner) pregnant, change in intention, no change in intention] and with the control variables specified in Part 1.1. A summary of all the covariates and numbers in the model is given in Appendix 1. The predicted probabilities, calculated for each variable at the sample mean of all other covariates, and confidence intervals of the model are displayed. Multilevel models were performed in Stata using survey weights.

## Results

### Aggregate trends in late intentions and the gap between women

Table 1 shows the proportion of women who wished to have a child in the age groups 35–39, 40–42 and 43–44 years, and also shows how this changed over time. The results reveal that the proportion of women who wished to have a child (or further children) increased strongly in all the age groups featured: women have postponed family formation such that an increasing proportion wish to have a child (or further children) at older ages. In particular, at 43–44 years of age, the proportion of all women who wished to have a child increased from 0.3% in 1986 to approximately 4% in recent years, while the chance of having a child at that age is extremely small. The proportion who wished to have a child (or further children) increased to an equivalent extent for all parities, but childless women were much more likely to wish to have a child than women with one child, who themselves wished to have another child more often than women with two or more children. At 40–42 years of age, the proportion of women wishing to have a child (or further children) increased from 1.3% in 1986 to 11.6% in 2016, and this was much higher among childless women (29.8% in the most recent period). At that age, one-third to one-half of women are no longer able to give birth to a live child (Leridon, 2004), so > 4% (11.6% x 1/3) of the population in 2016 and 10% (29.8% x 1/3) of childless women may not have a child although they declared that they wanted to have a child.

**Table 1:** Proportion of women who wished to have a child (or further children), by survey year, age group and parity.

	1986	1991	1996	2001	2006	2012	2016
Age 35–39 years							
Childless women	21.1	25.6	16.4	34.3	47.1	49.7	62.3
Women with one child	10.2	15.7	11.4	17.7	32.9	30.8	31.7
Women with more children	2.9	3.2	3.5	5.7	6.7	9.9	6.8
All parities	**6.5**	**9.9**	**7.5**	**13.4**	**21.0**	**24.4**	**24.4**
*N*	*1937*	*1439*	*1530*	*1706*	*943*	*784*	*575*

Age 40–42 years							
Childless women	3.5	6.0	9.1	14.3	-	21.2	29.8
Women with one child	1.3	1.6	2.5	2.8	-	8.5	14.9
Women with more children	0.9	0.2	1.3	1.0	-	3.8	3.6
All parities	**1.3**	**1.3**	**2.4**	**3.1**	-	**8.4**	**11.6**
*N*	*924*	*874*	*844*	*1003*	*579*	*574*	*393*

Age 43–44 years							
All parities	**0.3**	**0.7**	**1.4**	**2.9**	-	**4.1**	**3.6**
*N*	*708*	*621*	*524*	*618*	*437*	*422*	*326*

Source: Austrian Micro-Census surveys 1986–2016.

Note: the sample size was not large enough to decompose intentions at 43–44 years of age by parity. In 2006, only women aged ≤ 40 years were asked their intentions. As such, intentions are displayed by survey year and not by cohort.

Survey question: ‘Do you wish to have one or several (additional) child at some point in your life? Please count ongoing pregnancies’. Possible answers: ‘yes’, ‘no’ and ‘don’t know’. This table displays the proportion of women who answered ‘yes’ to this question.

The proportion of all women who were childless and did not wish to have children at 34–36 years of age oscillated between 7.3% and 10.4%, and this did not increase across the birth cohorts observed (Table 2). In parallel, the proportion of women who had not entered motherhood at that age continued to increase because of fertility postponement, so the proportion of women who were childless and wished to have a child at 34–36 years of age increased from 5.4% in the 1950–59 birth cohort to 13.1% in the 1970–74 cohort. The proportion of eventually childless women also increased strongly in these cohorts, from 13% to 19%, and these results suggest that fewer of the childless women who still wanted children at 34–36 years of age eventually had them. The gap between the proportion of eventually childless women and the proportion of childless women who did not wish to have a child (or a further child) at 34–36 years of age in the same cohorts, which broadly represents the proportion of women who were ‘unwillingly childless’, confirms this. This gap increased from 4.8% among women born in the 1950s to 11.7% among women born in the early 1970s. Even if some women may have changed their intention between their mid-30s and their mid-40s – when childlessness was calculated – this demonstrates an increasing aggregate contrast between women’s wishes and actual childbearing. Taking the oldest cohort as a benchmark, it is likely that at least 7% (11.7–4.8%) of women unintentionally forwent childbearing in the recent birth cohorts.

**Table 2:** Calculation of the gap between % eventually childless and % childless and not wishing to have a child at 34–36 years of age among women, by birth cohort.

	1950–59	1960–64	1965–69	1970–74
Total % childless at age 34–36 years of age	13.6	17.1	18.4	20.4
% childless and not wishing to have a child	*8.2*	*10.4*	*8.2*	*7.3*
% childless and wishing to have a child	*5.4*	*6.7*	*10.2*	*13.1*
% eventually childless (around 45 years of age)	13.0	14.6	16.6	19.0

Gap (% of all women ‘unwillingly’ childless)	**4.8**	**4.2**	**8.4**	**11.7**

*N* at 34–36 years of age	1973	911	893	507
*N* to calculate childlessness	5170	2043	2035	1329

Source: Austrian Micro-Census.

Note: % eventually childless was calculated in each cohort using the average proportion without children across the 40–49-years age group from the Micro-Censuses. ‘Missing’ and ‘don’t know’ were evenly distributed between women who wished to have a child and women who did not wish to have a child. The gap is the difference between the proportion of eventually childless women and the proportion of childless women saying that they did not wish to have a child at 34–36 years of age in each cohort.

Finally, in the same birth cohorts, the mean total number of children intended at 34–36 years of age was compared with the number of children (Table 3). Austrian women born in 1950–59 had 1.78 children per woman at 34–36 years of age, but those born in 1970–74 had only 1.42 children at that age. The total number of children intended at 34–36 years of age decreased to a lesser extent, from 2.01 to 1.86. This is notably explained because the additional number of children intended increased slightly. This left an increasing gap between late intentions and realization, which passed gradually from a deficit of approximately 0.14 children in the 1950–59 birth cohort to a deficit of 0.24 children in the 1970–74 birth cohort. Hence, in the earlier cohorts, women were falling short of their aggregate intended family size at 34–36 years of age by approximately 7%, but this was approximately 13% in the later cohorts. This confirms that the decrease in completed fertility across cohorts did not entirely reflect decreasing wishes in terms of family size.

**Table 3:** Calculation of the gap between final family size at 40–44 years of age and total number of children wished at 34–36 years of age among women, by birth cohort.

	1950–59	1960–64	1965–69	1970–74
Mean number of children wished at 34–36 years of age	2.01	1.94	1.92	1.86
of which: number of children at 34–36 years of age	*1.78*	*1.75*	*1.62*	*1.42*
of which: additional number of children wished	*0.23*	*0.25*	*0.31*	*0.50*
Final family size at 40–44 years of age	1.87	1.82	1.73	1.62

Gap (absolute)	**−0.14**	**−0.12**	**−0.19**	**−0.24**
Gap in % of children wished	**−6.7**	**−6.2**	**−9.9**	**−12.9**

*N* at 34–36 years of age	1973	911	893	507
*N* to calculate final family size	5170	2043	2035	1329

Source and note: as in Table 2. The gap is the difference between final family size and mean number of children wished at 34–36 years of age in a cohort.

### Age profile of fertility intentions and realization for women and men

**Fig. 1:**
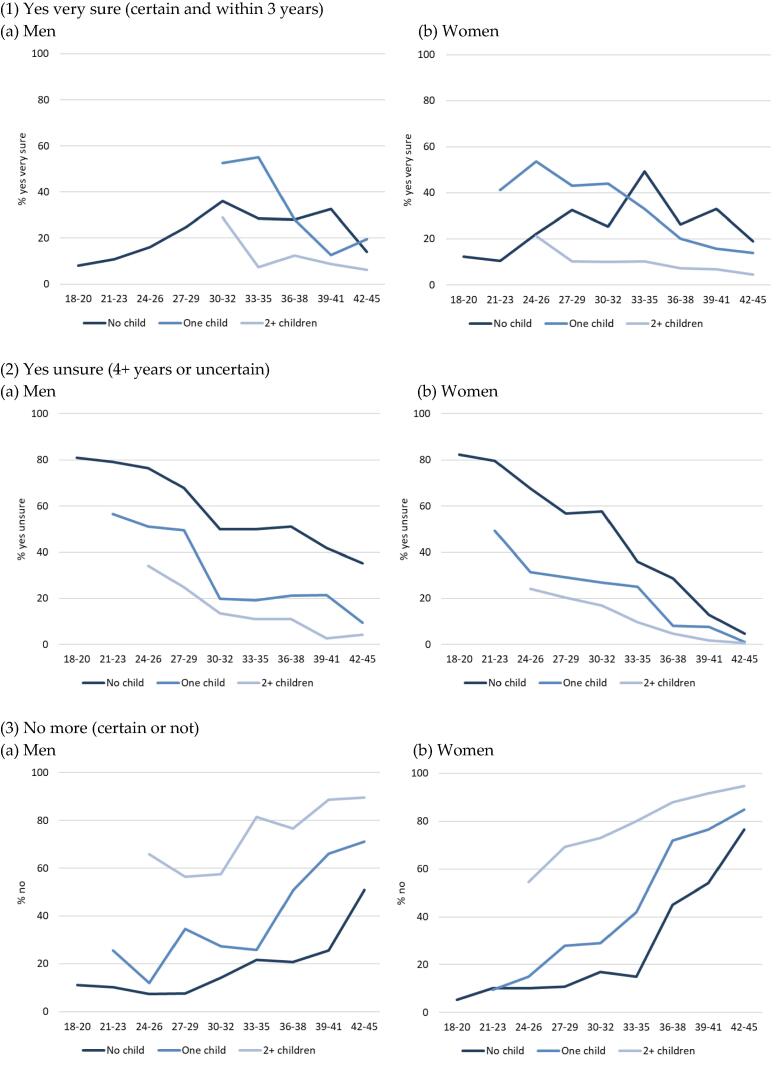
Percentage with a given intention in 2008/09, men and women by parity and age in 2008/09, Austria. Source: Austrian Generation and Gender Survey wave 1 (2008/09). Note: the number of observations available for the calculations is available in Table A2. The number of observations is generally large enough for the information displayed in these graphs to be reliable (mostly between 60 and 200, by age and parity). However, the information about men with one child is based on few observations (between 20 and 50) at most ages, and is therefore less reliable. Figures with confidence intervals are available on request.

At all ages and for both sexes, age-specific fertility intentions display extremely different levels depending on the number of children already born (Fig. 1). While the majority of young childless women were certain that they did not want a child in the next 3 years (Fig. 1.1), childless women who had reached their late 20 s up to late 30 s often did want a child in the next 3 years. The peak is at 50% for women aged 33–35 years. Childless men displayed an equivalent picture with a lower peak. Young women and men with one child were by far the most numerous to want a further child quickly (with an age shift for men, as they tend to have their children later). In contrast, a very low proportion of men and women with two or more children wished to have a further child compared with those with no children or one child. These intention profiles by parity mirror the general timing of births in Austria: a first child late, a second child soon afterwards, and rarely a third child.

The certainty of intentions also depends strongly on age and parity. A large proportion of young men and women reported that they wanted a child with less certainty (Fig. 1.2). This decreased almost linearly with age and to very low levels for women. The contrast was extremely strong by parity at young ages for both men and women: childless individuals were the most numerous to say that they wanted a child but without certainty, followed by those with one child, and finally those with two children or more. Differences between parities remained strong among men as they aged, but proportions converged towards zero among women of any parity. In parallel, negative intentions to have a child (or further children) increased in prevalence with age at any parity, the proportions being lowest among childless individuals and highest among those with two or more children (Fig. 1.3). Among childless women, the proportion who did not intend to have a child increased from 36 years of age onwards. The next section assesses whether this was because they had the children they intended to have, or – most probably – because they switched from a positive to a negative intention when reaching less fertile ages. Among childless men, the sudden increase took place later, at 42–45 years of age. Finally, both men and women with one child showed a gradual but ultimately strong increase in negative intentions with age.

**Fig. 2:**
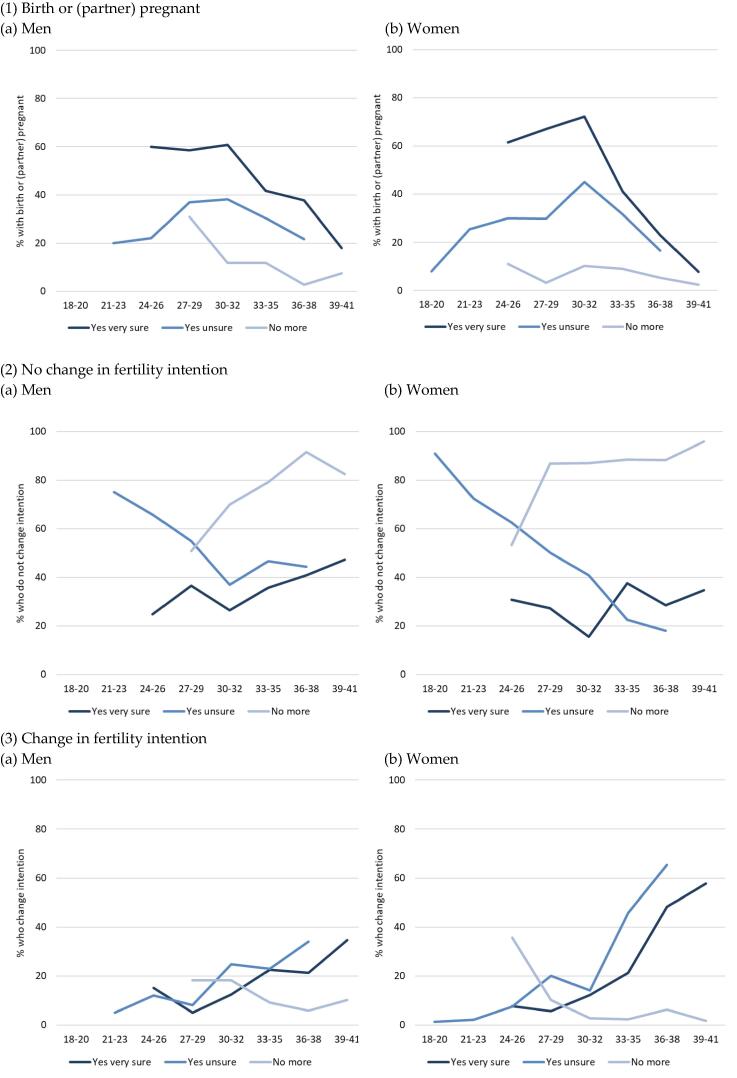
Childbearing, persistence or change in fertility intention between 2008/09 and 2012/13 depending on the original intention, by age in 2008/09 men and women, in percent. Source: Austrian Generation and Gender Survey wave 1 (2008/09) and wave 2 (2012/13). Note: the initial sample size, available in Table A3, is smaller than in Fig. 1 given the attrition between the waves. Among men of all ages together, the sample size is 284 for ‘yes, very sure’, 410 for ‘yes, unsure’ and 515 for ‘no more’ (369, 473 and 1089 for women, respectively). Information has not been displayed when less than 20 people answered the question, and the graph is stopped at 39–41 years of age for the same reason. In general, the sample in the cells displayed is approximately 40–70 answers, which is not very large, but the continuity across ages reinforces the reliability of the results. Figures with confidence intervals are available on request.

Fig. 2 shows what happened 4 years after the first wave, depending on the initial intention. The outcome depended on the initial intention as well as on age at first wave and sex. Those who had expressed a certain short-term intention were most likely to have had a child: up to 72% of women 30–32 years at the first wave and 61% of men (Fig. 1.2). However, among women aged > 32 years, this decreased so that < 10% of women aged 39–41 years in 2008/9 had a child by 2012/13. For men, the decrease was less steep, and one-fifth of them had the child they strongly intended to have by 40 years of age. Although women and men who were positive but uncertain about having a child were less likely to have a child, up to 40% of people in their early 30s had a child, and the frequency of realization tended to converge towards the frequency among the very certain in their late 30s. Finally, although they had said they did not want a child (certain or uncertain) in wave 1, a substantial proportion of men in their early 30s had a child in wave 2 (approximately 20% of those who had said ‘no’ in wave 1). Among women, the proportion remained < 10% and decreased more rapidly.

The proportion of men and women who persisted with their strong positive intention to have a child was between 20% and 40%, with values in the high range in individuals aged > 33 years, and even > 40% for men aged > 36 years (Fig. 2.2). Hence, despite lowered physiological perspectives on having children, women aged ≥ 33 years were at least as likely to persist with their strong positive intention as younger women; for men, this persistence seemed to increase (which will be checked in the following multivariate models). Women with positive but uncertain intentions were less numerous in that group over time, and a large proportion changed to a negative intention from 33 years of age (Fig. 2.3). While those with negative intentions before 30 years of age often had children or switched to a positive intention, women aged > 30 years and men aged > 36 years tended to keep their negative intention.

### Factors of realization, persistence or change within 4 years of strong short-term fertility intentions after 30 years of age

Table 4 features the predicted probabilities of the factors of having a child, of keeping the same intention or of changing intention by 2012/13 among men and women aged ≥ 30 years who expressed certain short-term fertility intentions in 2008/9, calculated in a multinomial regression. Realization of short-term positive fertility intentions declined strongly from 30 years of age for both men and women, although more strongly for women. More men and women changed their intention with age, and women aged > 36 years were particularly likely to change intention. Women and men aged > 33 years were less likely to have a child and more likely to persist with their intention than younger people. Childless women and men with no children or one child persisted with their strong positive intention to have a child, while parity was not significantly associated with the realization of that intention. Partnership status in the first wave was not significant among women, while single men less often had a child but were more likely to persist with their positive intention than partnered men. Finally, little association was found between level of education and realization or persistence of intention at these later ages. Only middle-educated women appeared to be less likely to have the child (or further children) they intended, and were more likely to persist with their positive intention than highly educated women, but this was at the limit of confidence intervals. In contrast, men with a university degree were more likely to persist with their strong positive intention than men with higher secondary education.

**Table 4:** Predicted probabilities to have a child, to keep the same intention and to change intention between 2008/09 and 2012/13; 30–41-year-old (a) men and (b) women who initially strongly intended to have a child (or further children), calculated in a multinomial model.

(a) Men							
		Birth or pregnant		No change in intention		Change in intention	
		Pred. prob.	95% CI	Pred. prob.	95% CI	Pred. prob.	95% CI
Age in 2008/09 (years)	30–32	62	(48–77)	26	(13–39)	12	(3–21)
	33–35	40	(23–57)	35	(17–54)	25	(8–41)
	36–38	41	(23–58)	39	(21–56)	21	(6–35)
	39–41	19	(8–30)	50	(34–66)	31	(15–47)
Parental status	No children	42	(30–54)	41	(29–54)	17	(7–26)
	1 child	41	(26–56)	40	(24–57)	19	(7–30)
	≥2 children	37	(15–60)	19	(2–36)	44	(19–68)
Partnership status	No partner	20	(5–34)	56	(36–75)	25	(7–42)
	Married	53	(40–65)	27	(16–37)	21	(10–32)
	Partner unmarried	55	(38–72)	33	(17–50)	12	(2–21)
Level of education	Low	41	(30–53)	38	(26–49)	21	(12–30)
	Medium	40	(21–60)	26	(9–44)	33	(15–51)
	High	42	(24–60)	47	(29–65)	11	(0–22)
Number of men		176		176		176	
Number of events		81		58		37	

(b) Women							
		Birth or pregnant		No change in intention		Change in intention	
		Pred. prob.	95% CI	Pred. prob.	95% CI	Pred. prob.	95% CI
Age in 2008/09 (years)	30–32	72	(57–86)	17	(4–30)	11	(2–20)
	33–35	43	(28–57)	38	(23–52)	20	(10–30)
	36–38	25	(10–40)	29	(11–47)	46	(25–67)
	39–41	8	(-2–19)	30	(12–47)	62	(43–81)
Parental status	No children	39	(25–52)	43	(30–57)	18	(8–28)
	1 child	35	(18–52)	25	(11–38)	40	(23–57)
	≥2 children	26	(10–41)	21	(5–37)	53	(34–72)
Partnership status	No partner	31	(15–47)	36	(19–54)	33	(17–49)
	Married	37	(23–50)	30	(18–43)	33	(20–46)
	Partner unmarried	43	(23–63)	31	(15–48)	26	(7–44)
Level of education	Low	37	(23–51)	31	(18–45)	32	(18–46)
	Medium	27	(12–42)	43	(25–61)	30	(14–46)
	High	42	(24–61)	27	(11–42)	31	(15–47)
Number of women		186		186		186	
Number of events		77		48		61	

Pred. prob., predicted probability; CI, confidence interval.

Source: Austrian Generations and Gender Survey wave 1 (2008/09) and wave 2 (2012/13).

Note: Sample size is given in Table A1; predicted probabilities are calculated by fixing the value of the other variables at their mean.

## Discussion

This exploration of late fertility intentions and late childbearing in Austria has brought about results of key importance for the understanding of fertility postponement and its consequences for reproduction. First, more and more people have not yet ‘completed’ their fertility when they reach less fertile ages, so that more of them intend to have a child after 35 years of age regardless of parity. Fertility at ≥ 35 years of age has increased but not sufficiently for people to catch up with their delay and to fulfil these later intentions. It was estimated that approximately 12% of all Austrian women born in 1970–74 wanted at least one child at 34–36 years of age but did not have any children, and that the final family size for this birth cohort was 13% smaller than the average intended family size at 34–36 years of age. In comparison with the 1950–59 birth cohort, the strong increase in excess childlessness and fertility gap from almost 5% to 12% and from 7% to 13, respectively, together with the strong increase in the proportion of women who wished to have a child (or further children) at 40–42 years of age (from 1% to 12% between 1986 and 2016), suggests that a substantial proportion of women may now unintentionally forego childbearing.

Further, age was confirmed as a central element of intentions and of realization. Intentions were extremely dependent on age, with a large proportion of ‘yes, unsure’ at young ages, of ‘yes, sure’ in the mid-30 s, and a large shift to ‘yes, unsure’ and ‘no’ afterwards. Given the narrow definition of strong positive intentions (certain and within 3 years), it was noticeable that even when an individual had strong intentions to have a child in the short term, this was realized by 72% of women and 62% of men of peak childbearing age (30–32 years) within 4 years, and much fewer individuals at most other ages (in descriptive as well as with predicted probabilities). First, fertility intentions remain too subjective to fully predict whether somebody is currently trying to conceive a baby, even with such a narrow definition. Second, even if the person is trying to conceive a baby at a given time, unexpected events can counteract intentions at all ages (e.g. partnership break-up, change in employment situation, etc.). From 30–32 years of age, realization in 2012/3 of the strong positive intention declared in 2008/9 decreased quickly for women to 8% at 38–41 years of age at the first wave and more slowly for men to 19% (again, in descriptive as well as with predicted probabilities). This shows that the realization of a strong desire to have a child is less likely at older ages than at younger ages. One can wonder why this is the case to such an extent. Indeed, the proportion of women actually having a child (or a further child) is well below their theoretical physiological ability at that age (approximately 55–65% of women trying to conceive at 40 years of age could have a live birth naturally). Such an age effect was also visible when only considering those persons in a couple (results available on request), so it cannot be explained by partnership status. The age effect also persisted after controlling for level of education, partnership status and parity. Late childbearing thus appears to be constrained beyond the biological limits, possibly by social barriers, normative age limits and life circumstances, and in a larger extent for women than for men.

These results indicate a gap between fertility intentions and realization, but they are not sufficient to affirm that all of these women unintentionally forwent childbearing. Establishing such a number is methodologically challenging for two reasons. First, fertility intentions are not set in stone and can change over the life course; in particular, they can be influenced by changes in life circumstances. Such variation was confirmed by this study, and notably, discontinuing a strong positive intention was particularly likely when nearing the last reproductive years. This supports the results of previous research and seems to be linked to short- and long-term adjustments of intentions to what seems realistic (Wagner et al., 2019). Whether this should be considered as surrendering or as a simple late switch is an important part of quantifying unrealized fertility, and more generally of understanding late fertility mechanisms. Second, intentions are uncertain, and answers to survey questions do not necessarily reflect the reality. Indeed, although fertility intentions are, to a certain extent, good predictors of childbearing behaviour, they are subject to strong uncertainty and are not necessarily well defined in people’s minds (Bachrach and Morgan, 2013, Kuhnt and Buhr, 2016, Ní Bhrolcháin and Beaujouan, 2019). Hence, a simple ‘yes’ to a fertility intention question is not sufficient to assess whether a person really wants a child, and more precise measures are necessary.

In addition, one cannot exclude increasing selectivity with age. Indeed, many women switched from a positive to a negative intention before reaching less fertile ages. This may correspond to self-selection into a group of intentions that best fits the perceived ability to have children and its likelihood – possibly a way for women to accept what will probably happen although it was not their first choice. Then, if women and men with the weakest fertility intentions, or who perceive their realization as unrealistic, leave the group of those with strong intention to have a child (or further children), those who remain in this group after their mid-30s are those with the strongest will to have children. On the other hand, this group probably includes more people with a lower propensity to have children and who therefore remained in this group, whereas others had children and therefore left the group. Overall, whichever effect dominates, women remaining in the group with strong short-term fertility intentions are less likely to have children regardless of age, down to 8% of births within 4 years at 39–41 years of age. Further studies are needed to explore the importance of the frequency of sexual intercourse for this age effect, given that it decreases with age and with couple duration (Frank et al., 1994). Dunson et al. (2004: 55) note, for instance, that ‘The effect on fertility of a man aging from 35 to 40 is about the same as the effect seen when intercourse frequency drops from twice per week to once per week’. It also acts perceptibly for women. Hence, the way that infertility and sexuality intertwine to explain childbearing at older ages deserves further investigation.

To address pitfalls in measurement and selection effects, the fertility gap was estimated in several different ways, including trends over time; overall, it was estimated that 4–7% of women in Austria in recent years had to forego the birth of a child (or a further child) that they would have liked to have. The surge in the number of people with positive intentions at later ages and in the number of children born suggests that an increasing number of people are trying to have children after 35 years of age, and potentially not managing. This corresponds with the observation that an increasing number of people are seeking help to procreate (Ben Messaoud et al., 2020, Goisis et al., 2020). In particular, the proportion of adults who do not eventually have the child(ren) they intended in their mid-30s increased quickly as fertility was postponed. These results highlight a strong contrast between the theoretical framework of fertility postponement and recuperation and the reality: in Austria, people who postpone having children ultimately have fewer children than they intended. Thus, the impact of fertility postponement on fertility levels should not be underestimated, and this has led to increasing demand for ART as people who delayed family formation in recent years start to try to have children. Studies in other countries and with different data or different definitions of fertility intentions are needed to test how resistant such results are to data, method and country.

The gap between the intention to have children and realization is expanding despite the increasing utilization and accessibility of ART. Although unrealized fertility may have been even more widespread in the absence of ART, ART does not fully compensate for declining fecundity with age (Leridon, 2004). People often think that they can use ART at later ages if they want children, but its success decreases with age unless one resorts to oocytes from younger donors or uses one's own oocytes frozen at a younger age (Beaujouan and Sobotka, in press, O’Brien and Wingfield, 2018). The context in which people try to access ART is also significant. Austria had particularly restrictive laws and norms for a long time until a more liberal Reproductive Medicine Law was implemented in 2014 (Griessler and Hager, 2016). In 2015, the proportion of ART infants among national births was slightly above the European average (2.9% versus 2.3%) (De Geyter et al., 2020). Nonetheless, public funding for ART treatments is only available up to 39 years of age for woman, while reimbursement is often offered beyond this age in other European countries (Calhaz-Jorge et al., 2020). After that age, women must meet the cost of all interventions themselves. The increase in the proportion of people who want a child at a later age and who potentially need to use ART raises awareness about the potential gap between accessibility of assisted reproduction and needs. People with greater resources and higher levels of education are more likely to use ART – even after adjustment for maternal age – even in the highly subsidised setting of Norway (Goisis et al., 2020). The lack of subsidies beyond 40 years of age in Austria may reinforce this social pattern of inequal access, and research on that topic is necessary. The current legislations on age limits to ART public funding could then be discussed again, carefully taking both sides of the argument into account: on the one hand, diminishing efficiency of ART with age and safety concerns for the mother and child; and on the other hand, increasing individual needs and risk of increased inequalities between those who are able to afford full ART costs at later ages and those who are not.

## Declaration

The author reports no financial or commercial conflicts of interest.

## Figures and Tables

**Table A1:** Number of men and women aged 30–41 years in 2008/09 available for the study of childbearing and change in intention between 2008/09 and 2012/13, Austria.

	Men	Women
Outcome		
Birth or pregnant	81	77
Change intention	37	61
Do not change intention	58	48
Age (years)		
30–32	57	55
33–35	44	55
36–38	34	36
39–41	41	40
Number of children		
0	87	69
1	58	68
≥2	31	49
Partnership status		
No partner	35	45
Married	97	95
Partner unmarried	44	46
Level of education		
Low	99	92
Medium	37	40
High	40	54
**Total**	**176**	**186**

Source: Austrian Generations and Gender Survey waves 1 and 2.

Sample: men and women aged ≥ 30 years in the first wave who expressed a strong positive intention to have children in the next 3 years or who said they wanted a child now (2008/09).

**Table A2:** Number of men and women aged 18–45 years available to calculate the percentage with a given intention in 2008/09 (Fig. 1), by age and parity.

(a) Men				
Age (years)	No child	One child	≥2 children	Total
18–20	64	2	0	66
21–23	178	5	1	184
24–26	157	26	6	189
27–29	144	23	21	188
30–32	90	46	43	179
33–35	81	44	65	190
36–38	74	53	96	223
39–41	65	57	139	261
42–45	64	65	181	310
**All**	**917**	**321**	**552**	**1790**

(b) Women				
Age (years)	No child	One child	≥2 children	Total
18–20	78	4	1	83
21–23	189	26	7	222
24–26	200	58	30	288
27–29	167	59	63	289
30–32	92	67	130	289
33–35	71	66	162	299
36–38	62	82	217	361
39–41	59	78	264	401
42–45	89	102	300	491
**All**	**1007**	**542**	**1174**	**2723**

Source: Austrian Generations and Gender Survey (GGS) wave 1.

Sample: men and women interviewed in the first wave of the Austrian GGS (2008/09).

**Table A3:** Number of men and women aged 18–45 years in 2008/09 available to calculate the realization of fertility intention between 2008/09 and 2012/13 depending on their original intention (Fig. 2), by age in 2008/09 and original intention.

(a) Men				
Age (years)	Certain now or within 3 years	Yes within ≥ 4 years or uncertain	No more (certain or not)	Total
18–20	4	22	5	31
21–23	9	82	9	100
24–26	28	87	12	127
27–29	39	73	21	133
30–32	57	43	37	137
33–35	44	29	57	130
36–38	34	36	85	155
39–41	41	18	131	190
42–45	28	20	158	206
All	284	410	515	1209

(b) Women				
Age (years)	Certain now or within 3 years	Yes within ≥4 years or uncertain	No more (certain or not)	Total
18–20	8	39	3	50
21–23	17	105	15	137
24–26	62	111	27	200
27–29	65	72	51	188
30–32	55	59	96	210
33–35	55	49	120	224
36–38	36	21	221	278
39–41	40	12	245	297
42–45	31	5	311	347
All	369	473	1089	1931

Source: Austrian Generations and Gender Survey waves 1 and 2.

Sample: men and women aged 18–45 years in the first wave (2008/09) and still present in the second wave (2012/13).
